# An Automated Vision-Based Inspection System for Metallic Lock Surface Defects Using a Transformer-Enhanced U-Net

**DOI:** 10.3390/s26092608

**Published:** 2026-04-23

**Authors:** Hong-Dar Lin, Shun-Yan Li, Chou-Hsien Lin

**Affiliations:** 1 Department of Industrial Engineering and Management, Chaoyang University of Technology, Taichung 413310, Taiwan; s11115613@gm.cyut.edu.tw; 2 Department of Civil, Architectural and Environmental Engineering, The University of Texas at Austin, Austin, TX 78712-0273, USA; chslin@utexas.edu

**Keywords:** metallic surface defect inspection, industrial machine vision, semantic segmentation, TransU-Net, deep learning

## Abstract

Surface defect inspection of metallic lock components remains challenging due to strong specular reflections, low-contrast defect patterns, and geometric variability, which limit the consistency of manual inspection and conventional automated optical inspection (AOI) systems. This study presents an integrated visual inspection framework that combines controlled image acquisition with deep learning-based semantic segmentation to enable reliable and repeatable defect detection. A standardized rotational fixture with ring illumination was developed to stabilize imaging geometry, reduce reflection variability, and support consistent multi-view acquisition. A region-of-interest (ROI) masking strategy was further applied to suppress background interference and isolate the effective inspection region. At the algorithmic level, a Transformer-enhanced U-Net (TransU-Net) architecture was employed to jointly model local spatial features and global contextual dependencies, thereby improving boundary delineation and the detection of irregular surface anomalies. In addition, a boundary-aware weighted evaluation scheme was introduced to provide a more robust and application-relevant assessment by accounting for annotation uncertainty near defect edges. Experimental results demonstrate that the proposed method achieved an F1-score of 85.15%, with an average inference time of 0.3357 s per image for model prediction. Considering additional processes such as multi-view image acquisition, mechanical rotation, and preprocessing, the overall system-level inspection time is expected to be on the order of seconds per component in practical deployment.

## 1. Introduction

Surface quality inspection is a critical component of modern manufacturing, directly affecting product reliability, customer satisfaction, and production efficiency. In high-volume metallic component fabrication, visual inspection remains widely used; however, manual inspection is inherently limited by human subjectivity, fatigue, and variability, leading to inconsistent quality control and limited scalability. Recent advances in computer vision and deep learning have enabled automated inspection systems that deliver more consistent, objective, and high-throughput defect detection, positioning intelligent visual inspection as a practical alternative for industrial quality assurance [1,2].

A pin tumbler lock is a mechanically robust locking mechanism widely used in industrial and commercial applications because of its structural simplicity and manufacturing efficiency. Its operation relies on multiple cylindrical pin stacks that must align at the shear line to enable plug rotation when the correct key is inserted. The lock examined in this study was primarily fabricated from a zinc alloy with a chrome-plated exterior, with some variants incorporating a stainless steel cap. These material choices provide favorable casting performance, corrosion resistance, and surface durability; however, the chrome-plated metallic surface exhibits strong specular reflection and high brightness variation. Such optical properties introduce imaging challenges, including glare, low defect contrast, and view-dependent appearance changes, which complicate reliable surface defect localization. Consequently, the lock’s material composition and geometry directly influence the illumination design, image acquisition strategy, and segmentation performance in automated vision-based inspection systems.

Lock products are manufactured in large volumes at low unit cost (approximately USD 2–5 per piece). At present, surface defect inspection is primarily performed through manual visual inspection, in which operators determine whether a lock should be accepted or rejected based on its surface appearance. However, due to the highly reflective nature of metallic surfaces, inspection must be conducted under strong illumination. The accuracy of manual inspection is strongly influenced by operator experience and visual fatigue, leading to frequent misjudgments. The current false detection rate is approximately 15–20%, while the missed detection rate is around 20–25%. In addition, inspection time per lock is about 5–10 s for experienced inspectors and 10–15 s for less experienced personnel.

The main challenges in lock surface defect inspection stem from the low visual contrast between defects and the surrounding surface, gradual intensity variations that produce gradient-like appearances, and the simultaneous presence of bright and dark defects with markedly different contrast characteristics. In addition, many defects have ambiguous or blurred boundaries, particularly for shallow surface defects, and exhibit significant shape variability, even within the same defect category. These factors collectively increase the difficulty of reliably localizing and segmenting defects on reflective metallic surfaces.

To address the challenges of low contrast, surface reflectivity, irregular defect morphology, and the limitations of manual inspection, this study proposes an automated surface defect inspection system for metallic locks based on computer vision and deep learning. The proposed framework integrates a customized image-acquisition setup, an ROI masking strategy, and a Transformer-enhanced U-Net (TransU-Net) architecture for robust semantic segmentation of complex surface defects. Unlike conventional defect inspection methods that rely primarily on algorithmic improvements, the proposed approach jointly considers imaging stability and model design by employing a standardized rotational fixture, controlled ring illumination, and ROI masking to reduce geometric variation and specular reflection before training. The TransU-Net architecture further enhances segmentation performance by combining convolutional feature extraction with Transformer-based global contextual modeling, enabling the accurate detection of low-contrast and irregular defects on reflective metallic surfaces. In addition, a boundary-aware weighted evaluation strategy is introduced to reduce the influence of annotation uncertainty and ambiguous defect edges, which are common in reflective materials. Experimental results demonstrate that the proposed system achieves reliable segmentation accuracy while maintaining practical computational efficiency, indicating its suitability for the real-world industrial inspection of small metallic components.

## 2. Literature Review

Surface defect inspection of metallic components has been extensively studied across manual inspection, classical AOI, and modern deep learning paradigms. Numerous surveys emphasize that although visual inspection remains common due to its flexibility, it suffers from subjectivity, operator fatigue, and inconsistent decision quality, limiting its suitability for high-throughput manufacturing environments [3,4,5,6]. Recent reviews further highlight that industrial defect inspection must address challenges such as reflective surfaces, low contrast, and irregular defect morphology, which complicate reliable automation [7,8,9]. These limitations motivate a transition toward vision-based, learning-driven inspection systems.

### 2.1. Conventional AOI Approaches

Traditional AOI techniques rely on handcrafted image-processing pipelines that include threshold segmentation, edge detection, morphological filtering, and engineered texture descriptors. Studies of metallic inspection show that these deterministic approaches perform adequately only under tightly controlled illumination and surface conditions [7,9]. However, rule-based pipelines are poorly robust when confronted with reflective artifacts, gradient intensity variations, or irregular defect boundaries, all of which are characteristic of metallic components [1,2].

Machine-learning research further indicates that handcrafted feature engineering lacks adaptability and generalization as defect variability increases [8,9]. Moreover, the scarcity of labeled industrial defect data continues to constrain the scalability of traditional inspection pipelines [4]. These findings collectively suggest that deterministic AOI frameworks are fundamentally limited in addressing the complex visual characteristics of metallic surfaces.

### 2.2. Optical Acquisition Challenges in Reflective Metallic Workpieces

Metallic surfaces pose imaging challenges due to specular reflection, glare, and viewpoint-dependent appearance. Optical inspection studies have explored illumination optimization, photometric stereo, and polarization imaging to enhance defect visibility [6,9]. Photometric stereo captures multi-directional lighting responses to infer surface geometry, enabling subtle defect highlighting, while polarization techniques suppress specular components to improve defect contrast. Reviews of industrial inspection systems emphasize that although such optical strategies improve defect observability, they often require complex acquisition setups that limit deployment on high-speed production lines [3,5]. These sensing limitations reinforce the need for integrated system designs that balance acquisition simplicity, repeatability, and algorithmic robustness, a recurring theme across multiple studies [4,8].

### 2.3. Deep Learning for Metallic Surface Defect Inspection

Deep learning has become the dominant paradigm for metallic surface inspection, offering superior feature representation and adaptability compared with handcrafted pipelines. Classification-based approaches provide a rapid assessment of defect presence but lack spatial localization [10]. Object detection frameworks, particularly YOLO-derived models, improve localization but struggle to capture irregular defect boundaries required for quantitative evaluation [11,12].

Semantic segmentation architectures, especially U-Net-derived frameworks, have shown strong performance in pixel-level defect mapping. Benchmark studies confirm that multi-scale contextual fusion improves segmentation accuracy under challenging illumination conditions [13,14,15]. Additional studies emphasize the importance of architectural enhancements such as residual learning, dense skip connections, and feature recalibration for improving defect representation [16,17]. Konovalenko et al. [18] systematically evaluated U-Net variants for metallic surface inspection, demonstrating that segmentation performance depends strongly on encoder–decoder feature balance and training stability.

Additional model innovations emphasize defect-specific representation learning. Wang and Xie [19] proposed a revised CNN architecture (MeDERT) to improve discriminative defect features, highlighting the importance of domain-aware architectural design in metallic inspection tasks. Efficiency-oriented models have also emerged; Yasir and Ahn [20] introduced channel-shuffling strategies to accelerate metallic defect detection while maintaining accuracy, addressing practical constraints in industrial deployment.

Recent studies further highlight the growing role of hybrid CNN–attention architectures in addressing the limitations of purely convolutional models [21,22]. Transformer-based segmentation frameworks provide improved global–local feature integration, which is essential for capturing irregular defect morphology and ambiguous boundaries [3,5]. At the same time, the literature consistently identifies limited labeled data and appearance variability as persistent challenges to generalization [4,8].

### 2.4. Persistent Challenges in Metallic Surface Defect Inspection

Despite these advances, four challenges remain widely acknowledged in the literature for metallic surface inspection: (1) Difficulty acquiring images caused by strong reflectivity and low-contrast defects [9,13]; (2) Boundary ambiguity, where shallow defects exhibit gradual transitions and lack clear contours, complicating pixel-level evaluation [14,15,18]; (3) The need for global–local feature fusion, since metallic defects often exhibit irregular shapes and fragmented appearances that are difficult to capture using purely convolutional features [3,16,19]; and (4) Data scarcity and generalization, which limit model robustness across production scenarios [4,8,20]. Multiple studies have concluded that effective inspection systems must jointly optimize sensing configuration, preprocessing strategy, segmentation architecture, and evaluation methodology rather than addressing these components independently [3,5,6].

Recent advances in AOI have explored both hardware-based and algorithm-driven approaches for surface defect detection. To address the limitations of traditional methods, convolutional neural network (CNN)-based models, such as U-Net and its variants, have been widely adopted for pixel-level segmentation. U-Net employs an encoder–decoder architecture for efficient feature extraction [23], while Res-U-Net improves gradient propagation through residual connections [24], and U-Net++ enhances feature fusion via dense skip connections [25]. Despite these improvements, CNN-based models remain limited in capturing long-range dependencies and global context. To overcome this limitation, attention-based and Transformer-integrated architectures have been introduced [26,27,28]. For example, TransU-Net incorporates self-attention mechanisms to model global relationships, enabling an improved representation of complex structures and irregular defect boundaries [26]. In parallel, hardware-oriented approaches, such as polarization imaging and multi-angle illumination, have been explored to mitigate specular reflection; however, these methods may introduce additional system complexity or reduce throughput in industrial environments.

Compared with existing approaches, the proposed method integrates controlled image acquisition and Transformer-enhanced segmentation to address both imaging and modeling challenges. The use of a rotational imaging setup and ROI masking reduces geometric and reflective variability at the data level, while the TransU-Net architecture enhances segmentation by capturing both local and global features. In addition, the proposed boundary-aware evaluation framework explicitly accounts for annotation uncertainty near defect edges, which was rarely considered in prior studies. This integrated design provides a more robust and practically applicable solution for defect inspection on reflective metallic surfaces.

While several recent studies have explored non-U-Net architectures, such as DeepLabV3+ [29], PSPNet [30], and Transformer-based models [31], these approaches are typically optimized for large-scale natural image datasets and rely on extensive training data and multi-scale context aggregation. Such characteristics may limit their applicability to small, high-resolution industrial datasets with subtle defect boundaries. In contrast, U-Net-based architectures remain the dominant choice in industrial surface defect inspection due to their effectiveness under limited-data conditions and their ability to preserve fine spatial details. Therefore, this study adopted U-Net and its variants as the primary comparison framework to ensure a fair and controlled evaluation. We acknowledge that comparisons with non-U-Net state-of-the-art architectures could provide additional insights; however, such models generally require substantially larger datasets and different optimization strategies, and are therefore beyond the scope of this study, which focused on controlled evaluation under limited-data industrial conditions.

## 3. Materials and Methods

This study presents an automated surface defect inspection framework for key locks that integrates controlled image acquisition, computer vision-based preprocessing, and deep learning-driven semantic segmentation. The proposed method is designed to segment surface defects at the pixel level accurately, enabling precise characterization of defect morphology and spatial extent while reliably distinguishing different defect categories. To ensure methodological clarity and reproducibility, the workflow is systematically organized into five sequential stages: image acquisition and preprocessing, manual annotation, model training, performance evaluation, and comparative analysis.

In the first stage, a dedicated image acquisition system is developed using a CCD camera, a ring-shaped illumination source, and an Arduino-controlled stepper motor integrated with a custom-designed 3D-printed rotational fixture. This configuration ensures stable sample positioning and precise angular control during image capture. The acquired images are batch-cropped to 450 × 450 pixels to suppress background interference and maximize the proportion of the lock surface within the field of view. Subsequently, circular and annular masks are constructed to extract the ROI, enabling downstream models to focus exclusively on surface defect regions.

In the second stage, the ROI images are binarized and color-inverted to enhance defect visibility, such that defect regions and edges are represented by high-intensity pixel values. Based on these enhanced images, pixel-level manual annotation is performed to generate ground-truth segmentation labels for each defect category.

In the third stage, multiple deep learning-based semantic segmentation models are developed and trained using the preprocessed images and their corresponding ground-truth labels. Systematic parameter tuning experiments are performed for each model to identify the optimal training configuration. After determining the optimal parameters, a self-attention mechanism is incorporated into the U-Net architecture, with the enhanced model retaining the same parameter settings as the baseline U-Net to ensure a fair comparison. Throughout the training process, overfitting is carefully monitored, and the optimal model weights along with relevant training statistics are retained for subsequent evaluation.

In the fourth stage, the trained models are applied to segment previously unseen test images using the saved optimal weights. Segmentation performance is quantitatively evaluated by computing category-wise metrics for each test image. In addition, the relative proportion of each defect category within an image is calculated to derive weighted performance metrics that account for defect distribution characteristics. Finally, in the fifth stage, the segmentation results of all evaluated deep learning models are systematically compared to assess their effectiveness and robustness in key lock surface defect inspection.

### 3.1. Description of Lock Samples and Defect Types

The sample used in this study was a pin-tumbler lock. The front of the cylinder (keyway) allows for direct visual observation of the internal pins, which is relevant for surface inspection of the lock head. The cylinder face has a diameter of 22 mm, and the chamfered surface height of the lock head is 6.12 mm. The internal mechanism uses a pin-seat design, resulting in relatively high assembly complexity compared with conventional mechanical locks. These structural characteristics and surface geometry make the lock suitable for evaluating automated surface defect inspection under reflective metallic conditions.

**Figure 1:**
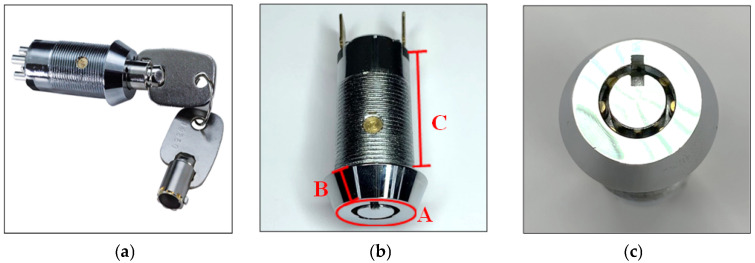
Visual illustration of a pin-tumbler lock sample: (**a**) assembled lock with key, (**b**) side view of the lock cylinder with surface regions labeled as A, B, and C, and (**c**) front view (Surface A) of the lock head.

Surface defects may occur on any part of a lock. Figure 1 illustrates the lock sample and the inspection regions defined by surface importance. As shown in Figure 1b, the lock surface is divided into three regions: Surface A, Surface B, and Surface C. Surface A corresponds to the front face of the lock head (keyway side), which is directly visible to the user and therefore represents the most critical inspection area. Surface B denotes the chamfered side region near the lock head and is considered secondary. Surface C corresponds to the threaded section of the cylinder body, which is typically concealed after installation and is therefore considered non-critical for visual quality. This classification was used in this study to prioritize defect analysis and evaluation.

**Figure 2:**
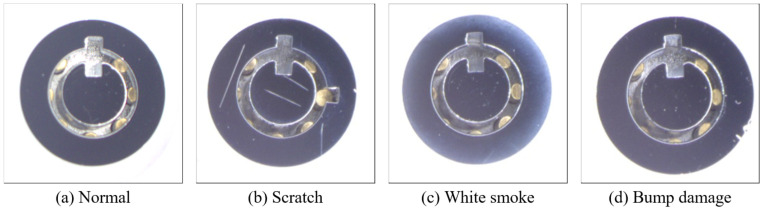
Representative examples of lock surface images for different defect categories: (**a**) normal (no defect), (**b**) scratch, (**c**) white smoke, and (**d**) bump damage.

Figure 2 presents representative samples of the four classes used in this study. The normal class contains defect-free surfaces, while the three defect categories include scratch, characterized by linear surface marks; white smoke, which appears as cloudy or hazy regions on the metallic surface; and bump damage, which manifests as localized protrusions or impact marks. These images illustrate the visual diversity and subtle appearance differences among defect types, highlighting the challenges of surface defect inspection on reflective metallic materials. In the case company’s production process, the defect rate for lock head surfaces is approximately 13% of total production, and the three defect types investigated in this study account for nearly 80% of all recorded defects.

In this study, surface defects were categorized into three types: scratch, white smoke, and bump damage. Each defect type may appear in two visual forms: bright and dark. Bright defects typically appear as light-colored surface artifacts, such as minor scratches and white smoke. In contrast, dark defects are usually associated with deeper surface indentations, such as severe scratches and bump damage. Due to the reflective nature of metallic materials, dark defects are often difficult to detect with the naked eye and usually require rotating the surface and observing under reflected light to become visible.

### 3.2. Image Acquisition of Lock Components

**Figure 3:**
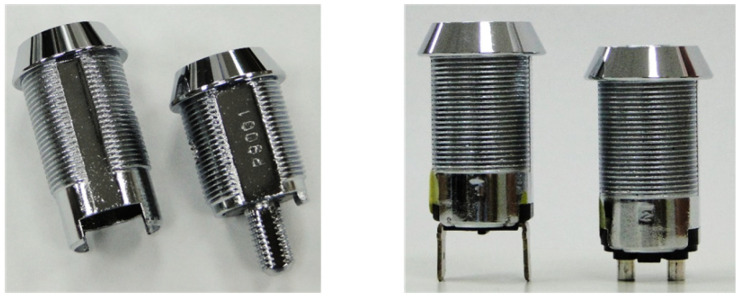
Structural variations among lock samples requiring a standardized fixture support for consistent imaging.

To obtain geometrically consistent lock images for segmentation, a controlled laboratory imaging setup was established. Lock assemblies have application-dependent base geometries and varying axial lengths, and certain configurations lack sufficient support features to maintain a stable upright posture. These mechanical variations introduce positional and height inconsistencies that directly affect the imaging geometry (Figure 3). To eliminate these mechanical and positional variabilities, a standardized holding fixture was engineered to accommodate multiple lock configurations. The fixture constrains the lock in a repeatable vertical orientation, compensates for dimensional differences, and establishes a fixed reference height relative to the imaging system, thereby ensuring consistent acquisition conditions across all samples.

**Figure 4:**
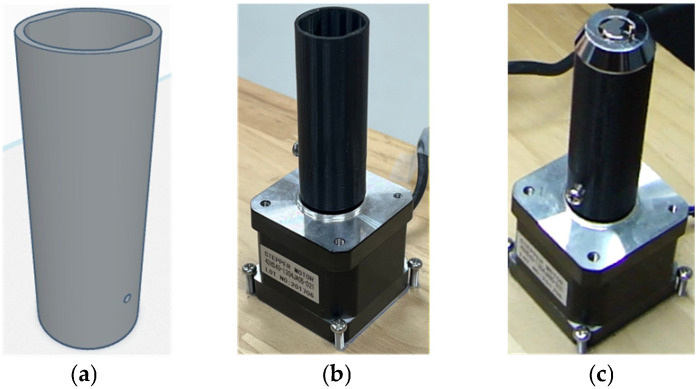
Design and implementation of the rotational holding fixture: (**a**) CAD model of the cylindrical fixture; (**b**) 3D-printed fixture mounted on the motor platform; (**c**) lock sample installed in the fixture for controlled rotation during image acquisition.

Figure 4 illustrates the development and practical implementation of the rotational holding fixture used to stabilize lock samples during imaging. Figure 4a presents the CAD model of the cylindrical fixture designed to securely constrain the lock body while maintaining geometric alignment. Figure 4b shows the fabricated fixture mounted on a motorized platform, enabling controlled rotational motion for multi-angle image capture. Figure 4c demonstrates a lock sample positioned within the fixture, highlighting how the assembly provides repeatable orientation and consistent imaging conditions. This fixture design minimizes positional variability and ensures stable sample handling during automated visual inspection.

To further reduce environmental and positioning variability, the fixture was integrated with an Arduino-controlled stepper motor and a dedicated control routine to precisely regulate the rotational angle during image acquisition. This motorized configuration ensures that each lock maintains a repeatable spatial position and orientation throughout capture. Because the inspected lock surfaces are circular, the rotation sequence was discretized into 90° increments, enabling uniform multi-view sampling. Consequently, four images were acquired per lock, providing consistent angular coverage while preserving geometric repeatability across the dataset.

**Figure 5:**
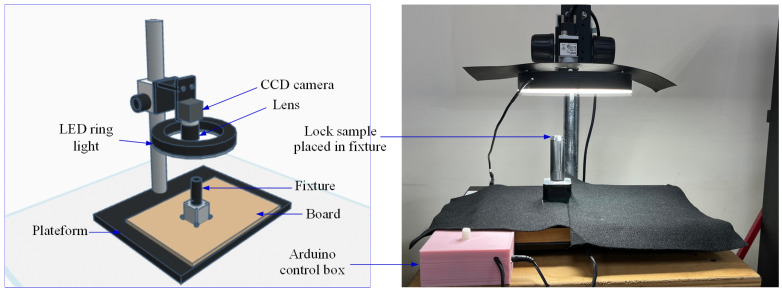
Image acquisition hardware configuration: schematic diagram and photograph.

The hardware used for image acquisition in this study included a light source controller, a 1.3-megapixel color CCD camera, a 10 mm lens, and an LED ring light. The image acquisition software interface was Vision Builder AI 2014 SP1. The experimental setup parameters were configured so that the distance between the ring light and the base was 17.5 cm, and the distance between the lock surface and the lens was 6 cm. The light intensity was set to 111 on a scale of 0–255, and the illuminance measured using a spectrometer was 1450.99 lux. Since the samples were metallic and highly reflective, black shielding paper was placed between the ring light and the lens to prevent reflections from surrounding objects from reaching the lock surface, which could otherwise interfere with defect visualization, as shown in Figure 5.

### 3.3. Image Preprocessing

**Figure 6:**
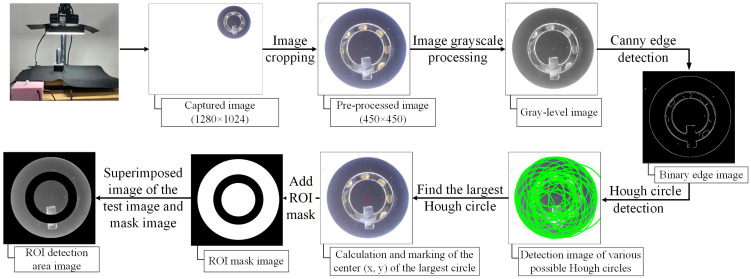
Procedures for extracting ROI regions for lock images.

To accurately localize the surface regions where defects are most likely to occur, an ROI extraction procedure based on annular masking was adopted, as illustrated in Figure 6. During image acquisition, the lock sample was deliberately positioned slightly off the optical center of the ring illumination to suppress specular reflections. The original images were captured at 1280 × 1024 pixels and subsequently cropped to 450 × 450 pixels to ensure that the lock occupied the majority of the image frame. The cropped image was first converted to grayscale, followed by Canny edge detection to extract prominent structural boundaries. The Hough circle transform was then applied to identify multiple candidate circular contours, from which the largest circle corresponding to the lock’s outer boundary is selected. The center coordinates of this circle were computed and used to generate concentric annular and outer circular masks. These masks were superimposed onto the original image to remove irrelevant background regions and internal components, retaining only the lock surface area of interest. The resulting ROI image was subsequently used for defect segmentation, allowing the model to focus exclusively on surface defects while minimizing background interference.

**Figure 7:**
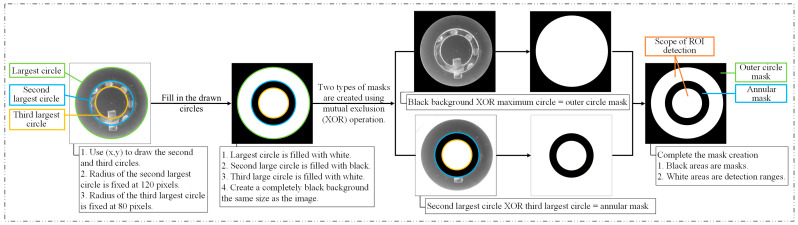
Procedures for creating ROI masks for lock images.

Figure 7 illustrates the detailed procedure for generating the ROI masks. First, the largest circle and its center are detected using the Hough Circle Transform. Based on the identified center, the second and third-largest circles are determined. Because the positional deviation of the rotating fixture is minimal, the radii of these circles can be directly set to 120 pixels and 80 pixels, respectively. The outer circle mask is obtained by applying a logical XOR operation between a full-black background (the same size as the image) and the largest white circle. The annular (ring-shaped) mask is generated by applying a logical XOR operation between the black second-largest circle and the white third-largest circle. The final mask used in this study was obtained by combining the outer circle mask with the annular mask. By overlaying these masks onto the lock image, the ROI-extracted image is obtained.

### 3.4. Generation of Label Images for Lock Images

**Figure 8:**
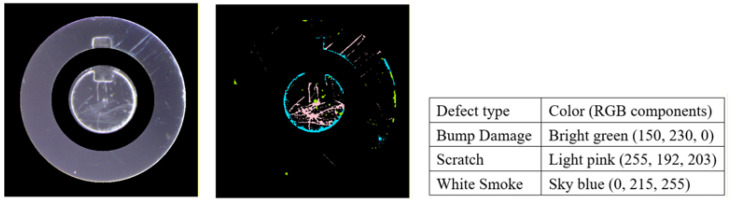
Example testing image, corresponding pixel-level labeled image, and RGB color encoding definitions for each defect category. Specifically, bump damage is represented in bright green (150, 230, 0), scratches in light pink (255, 192, 203), and white smoke in sky blue (0, 215, 255).

The deep learning semantic segmentation models used in this study require pixel-level annotations for training. Therefore, three software tools were employed sequentially to complete the labeling process. In the first stage, National Instruments IMAQ Vision Builder 6 is used. The ROI lock images are imported and processed by extracting the RGB channels, followed by convolution-based enhancement to highlight defect details. The images are then binarized, and the foreground and background are inverted to complete the initial preprocessing. In the second stage, Adobe Photoshop is used to overlay the original images with the binarized outputs generated in the first stage. Specific RGB colors are assigned to each defect category, and pixel-level annotations are manually applied to each binarized image for each defect type. In the third stage, a Python program (version 3.7.12, 4 September 2021) converts pixel values and resizes images to ensure consistency with the model’s input requirements. Figure 8 shows an example testing image, the corresponding pixel-level labeled image, and the RGB color-encoding definitions for each defect category (bump damage, scratch, and white smoke).

### 3.5. Semantic Segmentation Deep Learning Models for Lock Surface Defects

In this study, the preprocessed images and corresponding label images with 450 × 450 pixels were first resized to 256 × 256 pixels for model training. The rescaled metallic lock images were then used to train several modified U-Net–based architectures. After training and validation, the optimal parameter settings and model weights for each architecture were saved, and the trained models applied to segment unseen test images to verify the effectiveness of the selected parameter configurations. For the self-attention-enhanced model, the same parameter configuration as the baseline U-Net was adopted to ensure fair comparison. Finally, the segmentation performance of all models was quantitatively evaluated and compared using the defined performance metrics.

#### 3.5.1. U-Net Model Structure for Defect Segmentation of Lock Surfaces

**Figure 9:**
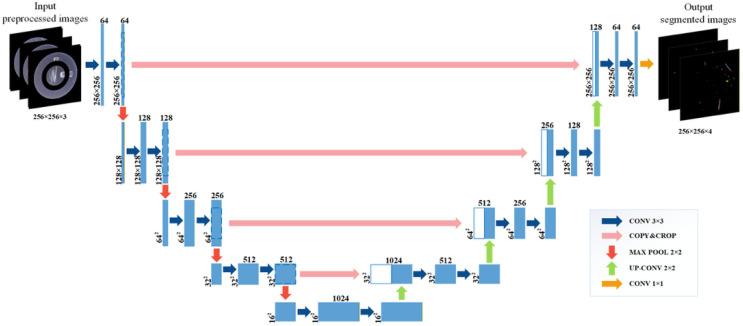
U-Net-based architecture for lock surface defect inspection.

U-Net [23] is a fully convolutional encoder–decoder network designed for pixel-wise semantic segmentation, featuring symmetric skip connections that fuse multi-scale contextual features from the encoder with high-resolution spatial information in the decoder. As illustrated in Figure 9, the architecture adopted in this study consists of five encoding–decoding stages tailored for lock surface defect segmentation.

In the encoder path, each stage contains two consecutive 3 × 3 convolutional layers followed by a 2 × 2 max-pooling operation for spatial downsampling. Zero-padding is applied before each convolution to maintain spatial dimensions within the same stage, so that the feature map resolution remains unchanged while the channel depth increases progressively (64 → 128 → 256 → 512 → 1024). The max-pooling layers reduce the spatial resolution by a factor of two at each stage, enabling hierarchical feature abstraction.

In the decoder path, spatial resolution is progressively restored using 2 × 2 up-convolution (transpose convolution) operations. The upsampled feature maps are concatenated with their corresponding encoder feature maps via skip connections, facilitating the integration of coarse semantic information with fine-grained spatial details. Each concatenation is followed by two 3 × 3 convolutional layers for feature refinement. Finally, a 1 × 1 convolution layer maps the decoded feature representations to the required number of output channels for defect category segmentation.

#### 3.5.2. Res-U-Net Model Structure for Defect Segmentation of Lock Surfaces

**Figure 10:**
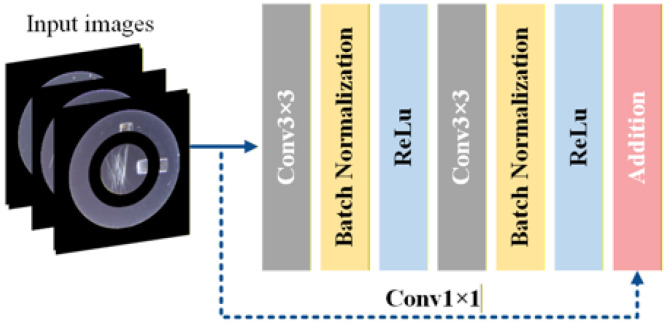
Architecture of the residual model (Res-U-Net block). Gray blocks represent convolutional layers (Conv 3 × 3), yellow blocks denote batch normalization, blue blocks indicate ReLU activation, and the red block represents element-wise addition with the shortcut connection. The dashed path corresponds to the 1 × 1 convolution used for channel matching in the residual branch.

Res-U-Net [24] extends the conventional U-Net architecture by integrating residual learning mechanisms into each convolutional stage to enhance feature propagation and improve training convergence. As illustrated in Figure 10, each residual block consists of two consecutive 3 × 3 convolutional layers, each followed by batch normalization and ReLU activation. In parallel to the main convolutional path, a shortcut branch employing a 1 × 1 convolution is introduced to match the channel dimensions. The output of this shortcut branch is element-wise added to the output of the main branch, forming a residual mapping that facilitates gradient backpropagation and preserves low-level structural information.

**Figure 11:**
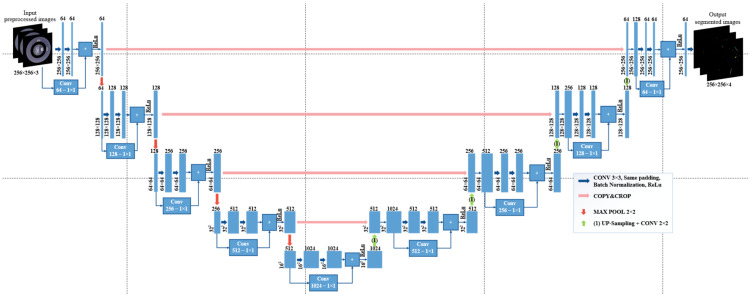
Res-U-Net-based architecture for lock surface defect inspection. Blue arrows indicate the flow of feature maps through the network, including convolutional layers and skip connections. Blue blocks represent convolutional operations (Conv 3 × 3) with batch normalization and ReLU activation. Red arrows denote max pooling for down-sampling, while green arrows indicate up-sampling operations in the decoder. The skip connections enable feature fusion between the encoder and decoder stages.

As shown in Figure 11, these residual blocks replace the standard convolutional units in both the encoder and decoder paths of the U-Net framework. Similar to the baseline architecture, zero-padding is applied before each 3 × 3 convolution to maintain spatial resolution within each stage, ensuring that feature map dimensions remain unchanged prior to pooling or upsampling operations, while only the channel depth is adjusted. Downsampling is performed using 2 × 2 max-pooling layers, and upsampling is achieved via up-convolution or transpose convolution layers. Skip connections are retained to concatenate encoder features with decoder features at corresponding scales. By incorporating residual connections throughout the network, the ResU-Net architecture reduces vanishing gradient effects, stabilizes deep feature learning, and improves segmentation performance for complex lock surface defect patterns.

#### 3.5.3. U-Net++ Model Structure for Defect Segmentation of Lock Surfaces

**Figure 12:**
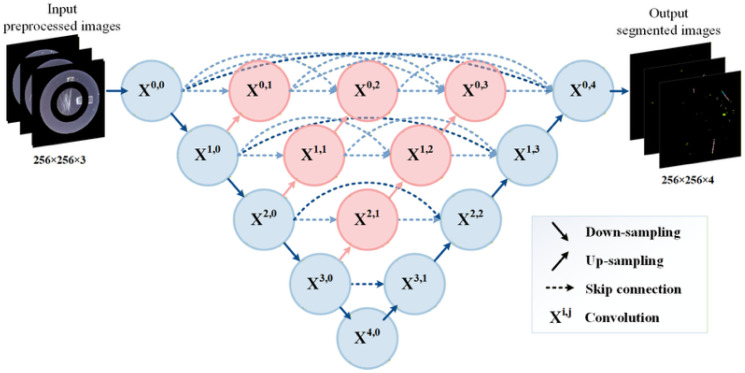
U-Net++-based architecture for lock surface defect inspection. Blue nodes represent feature maps along the encoder–decoder backbone, while red nodes denote intermediate dense skip-connection nodes that progressively fuse multi-scale features. Solid arrows indicate down-sampling and up-sampling operations, and dashed arrows represent skip connections.

U-Net++ [25] is a nested encoder–decoder architecture that extends the conventional U-Net by introducing redesigned dense skip connections to progressively bridge the semantic gap between encoder and decoder feature maps. As illustrated in Figure 12, the network is organized into a series of convolutional nodes denoted as X^i,j^, where i represents the depth level and j denotes the stage along the dense skip pathway.

Unlike the original U-Net, which directly concatenates encoder feature maps with the corresponding decoder features at the same scale, U-Net++ inserts intermediate convolutional nodes along each skip pathway. These nested dense connections enable multi-level feature aggregation before fusion with the decoder, thereby enhancing feature refinement and improving gradient flow across network depths. Each node in the nested structure performs a convolutional transformation to progressively align high-resolution spatial features with semantically enriched representations from deeper layers.

Downsampling is conducted along the encoder path through successive pooling operations, while upsampling in the decoder path is achieved using transposed convolution to restore spatial resolution. By densely connecting intermediate feature maps across different semantic levels, U-Net++ achieves more effective multi-scale feature fusion and improved segmentation performance for complex lock surface defect patterns.

#### 3.5.4. TransU-Net Model Structure for Defect Segmentation of Lock Surfaces

TransU-Net [26] is a hybrid encoder–decoder segmentation architecture that integrates Transformer-based self-attention into the U-Net framework to jointly model local spatial features and long-range global dependencies. While Attention U-Net [27] enhances the original U-Net by introducing attention gates within skip connections to selectively emphasize relevant encoder features, primarily improving local feature filtering, TransU-Net embeds a Transformer module at the bottleneck to explicitly capture global contextual relationships. This design enables more coherent segmentation of irregular, low-contrast, and spatially discontinuous structures.

**Figure 13:**
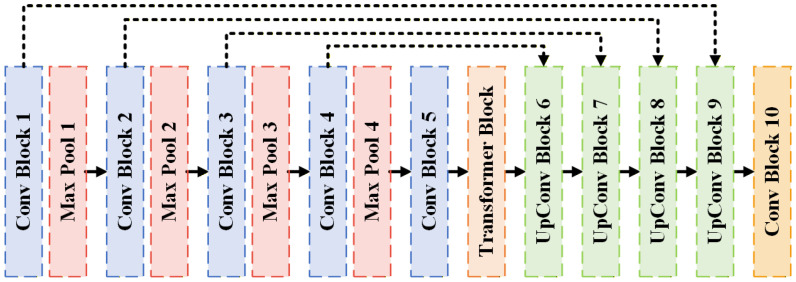
Overall architecture of the TransU-Net model. Blue blocks denote convolutional encoding stages, red blocks represent max-pooling operations for down-sampling, orange blocks indicate the Transformer module at the bottleneck, green blocks correspond to up-convolution (UpConv) decoding stages, and the final yellow block represents the output convolution layer. Solid arrows indicate the forward flow of feature maps through the network, while dashed arrows denote skip connections between encoder and decoder stages.

As illustrated in Figure 13, the proposed TransU-Net retains the standard multi-stage encoder–decoder structure of U-Net, consisting of successive convolutional blocks and max-pooling operations in the encoder, followed by symmetric upsampling stages in the decoder. The key modification lies in the insertion of a Transformer block between the encoder and decoder at the bottleneck layer. Specifically, the output feature map from the final encoder stage is first processed by an additional convolutional layer and reshaped from a two-dimensional feature map into a one-dimensional token sequence. Positional embeddings are then added to encode spatial information lost during reshaping. The token sequence is passed through multiple Transformer layers composed of multi-head self-attention and feed-forward networks to model global contextual interactions and long-range dependencies. After attention processing, the sequence is reshaped back into a two-dimensional feature representation and forwarded to the decoder for progressive upsampling and segmentation.

**Figure 14:**
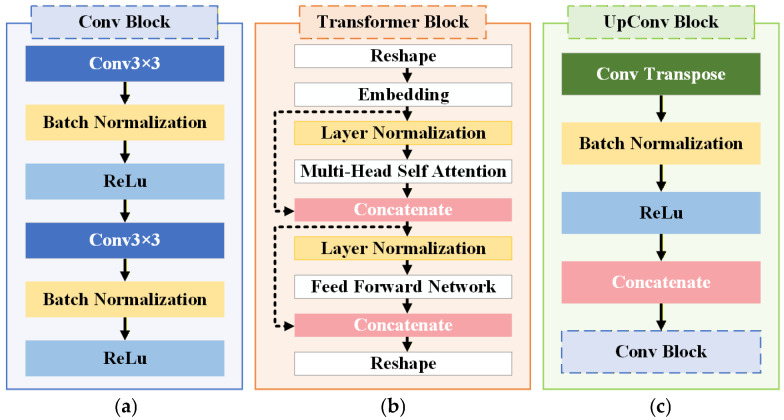
Structural components of TransU-Net: (**a**) Conv Block, (**b**) Transformer Block, and (**c**) UpConv Block used in the proposed encoder–decoder architecture. Colored blocks denote different operations, including convolution (blue), batch normalization (yellow), ReLU activation (light blue), reshape and embedding operations (white), Transformer processing layers (beige), concatenation (pink), and transposed convolution (green). The light green background indicates the grouping of UpConv modules. Solid arrows represent the forward flow of feature processing, while dashed arrows denote residual or skip connections within the Transformer block.

Figure 14 details the structural components of the TransU-Net architecture. The Conv Block (Figure 14a) consists of two successive 3 × 3 convolutional layers, each followed by batch normalization and ReLU activation, enabling hierarchical local feature extraction. The Transformer Block (Figure 14b) includes reshape and embedding operations, layer normalization, multi-head self-attention, residual feature concatenation, and a feed-forward network, forming a standard Transformer encoder structure adapted for dense prediction tasks. The UpConv Block (Figure 14c) employs transposed convolution for spatial upsampling, followed by batch normalization and ReLU activation. The upsampled features are concatenated with corresponding encoder features via skip connections and further refined using a Conv Block.

Overall, as shown in Figure 13, the proposed architecture comprises five convolutional stages in the encoder, a Transformer-based bottleneck for global representation learning, and symmetric decoder stages with skip connections to recover fine-grained spatial details. By integrating convolutional inductive bias with self-attention-based global modeling, the TransU-Net architecture effectively enhances segmentation robustness for complex metallic lock surface defects characterized by reflectivity, low contrast, and irregular morphology.

#### 3.5.5. FRCU-Net Model Structure for Defect Segmentation of Lock Surfaces

To further examine whether alternative attention mechanisms can achieve improved effectiveness–efficiency trade-offs compared with Transformer-based global modeling, this study evaluated the FRCU-Net model [28], which incorporates a Frequency Recalibration Unit (FRU) into the U-Net framework. Unlike TransU-Net, which captures long-range dependencies through spatial self-attention, FRCU-Net enhances feature representation in the frequency domain, aiming to strengthen texture and structural discrimination while maintaining a relatively lightweight architecture.

**Figure 15:**
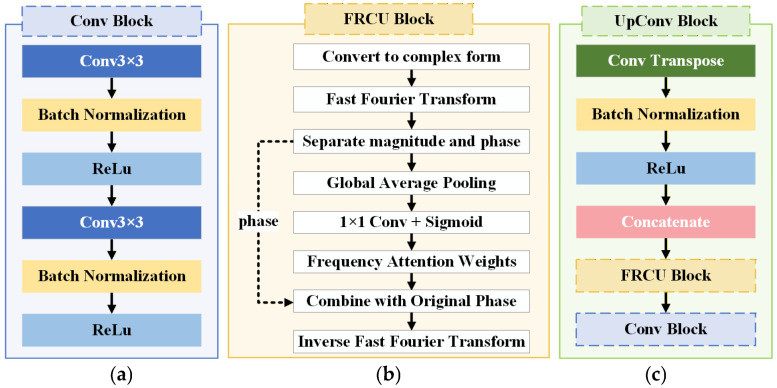
Structural blocks of the FRCU-Net model: (**a**) convolution block, (**b**) FRCU block, and (**c**) up-convolution block. Colored blocks denote different operations, including convolution (blue), batch normalization (yellow), ReLU activation (light blue), intermediate frequency-domain pro-cessing steps (white), concatenation (pink), and transposed convolution (green). The light green background indicates the grouping of UpConv modules. Solid arrows represent the forward flow of feature processing, while dashed arrows denote the propagation of phase information and in-ternal connections within the FRCU block.

As illustrated in Figure 15b, the FRU block operates by transforming the input feature map into the frequency domain using the fast Fourier transform (FFT). The resulting complex-valued spectrum is decomposed into magnitude and phase components. Global average pooling is applied to the magnitude component to extract global frequency descriptors that summarize channel-wise spectral responses. These descriptors are then passed through a learnable 1 × 1 convolution layer followed by a sigmoid activation to generate channel-wise frequency attention weights. The original magnitude spectrum is recalibrated using these learned weights to emphasize informative frequency components associated with salient texture patterns and suppress less relevant spectral responses. The recalibrated magnitude is subsequently recombined with the original phase information, and the inverse FFT (IFFT) is applied to transform the feature representation back into the spatial domain. The real-valued output is used as the refined feature map for subsequent decoding operations. This design enables the explicit modeling of spectral saliency while preserving spatial structure, thereby enhancing sensitivity to subtle, low-contrast, and texture-related defect patterns.

**Figure 16:**
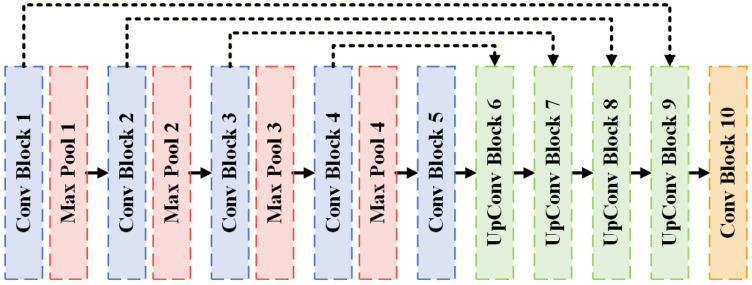
Complete architecture of the FRCU-Net model. Colored blocks indicate different functional modules, including convolution blocks (blue), max-pooling layers (red), up-convolution blocks (green), and the final convolution layer (orange). Solid arrows denote the forward propagation of feature maps, while dashed arrows represent skip connections between encoder and decoder stages for multi-scale feature fusion.

As shown in Figure 16, the overall topology of FRCU-Net remains structurally consistent with the standard U-Net encoder–decoder architecture. The modification is confined to the decoder pathway. Specifically, as illustrated in Figure 15c, the FRU module is embedded within the up-convolution block. During decoding, the upsampled feature maps are first concatenated with the corresponding encoder features via skip connections. The fused features are then processed by the frequency-domain recalibration module before being refined by the convolutional block shown in Figure 15a. This integration enables spectral attention to operate on multi-scale fused representations, improving reconstruction quality and defect boundary delineation.

## 4. Experiments, Results, and Discussion

### 4.1. Environment Development of the Detection System

A total of 1200 lock samples were randomly collected from the production lines of a lock manufacturing company in Taiwan for hyperparameter configuration and performance evaluation. The inspection system was developed in Python and deployed on a workstation equipped with an Intel Core i7-4790 CPU, 16 GB of RAM, an NVIDIA GeForce RTX 3060 GPU, and Windows 11.

**Figure 17:**
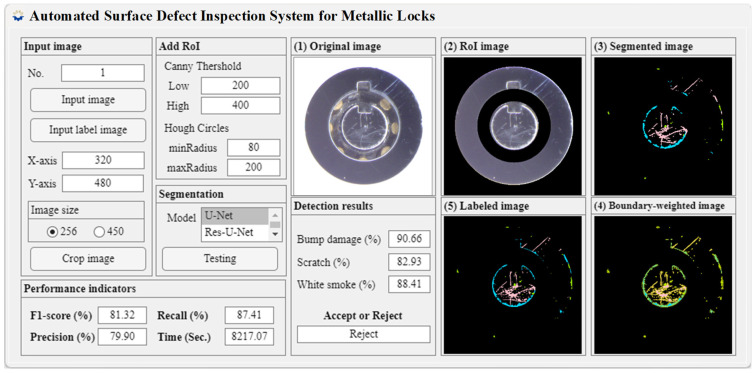
User interface of the detection system developed in this study. The color encoding of defect categories in the segmented and labeled images is consistent with that defined in Figure 8.

The preprocessed images, originally at 450 × 450 pixels, were rescaled to 256 × 256 pixels (1:1 aspect ratio). This resizing step was performed to reduce computational cost, improve training efficiency, and ensure consistent input dimensions for the deep learning models. Despite the reduction in resolution, the resized images preserved sufficient defect details for accurate segmentation. Predefined circular and annular masks were applied to suppress internal structural components, retaining only the ROI for defect analysis. Pixel-level annotation was performed to generate ground-truth labels for supervised training. After training and selecting the optimal model weights, segmentation was conducted on the test images, and quantitative performance metrics were computed. The proportions of detected defect regions were then used to determine acceptance or rejection decisions. As illustrated in Figure 17, the developed graphical user interface integrates all processing modules, providing a complete and automated inspection workflow for metallic lock surface defect detection.

### 4.2. Evaluation Indicators for Detecting Surface Defects in Metal Lock Cylinders

In this study, deep learning models were used to segment metallic lock images. The confusion matrix was employed to analyze the segmentation performance of the models by indicating whether the network correctly classifies each pixel in the image. The following evaluation metrics were adopted for performance analysis in this study: Intersection over Union (IoU), Dice coefficient (F1-score), Precision, and Recall.

#### 4.2.1. Calculation of Performance Metrics for Each Defect Category

To compute the classification performance metrics for each defect category within an image, a mask must first be generated. During evaluation, each input image and its corresponding ground-truth label image are processed sequentially. For both the input and label images, pixels corresponding to each defect category are extracted using predefined color settings, and performance metrics are then calculated from the generated masks. Let C = defect category; C_1_ = “Bumpdamage”; C_2_ = “Scratch”; C_3_ = “Whitesmoke”. Ground-truth label mask: label_mask (x, y), where the pixel at position (x, y) matches the color corresponding to the defect category. Predicted segmentation mask: pred_mask (x, y), where the pixel at position (x, y) matches the color corresponding to the defect category.

In a confusion matrix, the true condition corresponds to the ground-truth label, and the predicted outcome corresponds to the model’s prediction. Each of these has two possible states: Positive and Negative. Combining these outcomes yields four outcomes: True Positive (TP), False Positive (FP), True Negative (TN), and False Negative (FN). Based on these quantities, the following performance metrics are computed:

(1) IoU (Intersection over Union): The proportion of correctly detected pixels among all pixels in the union of the predicted and ground-truth regions, as shown in Equation (1):

(1) $${IoU}_{C_{n}}\%=\frac{TP}{TP+FP+FN}\times 100\%$$

(2) Dice coefficient (F1-score): Measures the similarity between the segmentation result and the ground-truth annotation, as shown in Equation (2):

(2) $${Dice}_{C_{n}}\%=\frac{2TP}{2TP+FP+FN}\times 100\%$$

(3) Precision: The probability that a pixel predicted as defective is truly defective, as shown in Equation (3):

(3) $${Precision}_{C_{n}}\%=\frac{TP}{TP+FP}\times 100\%$$

(4) Recall: The proportion of truly defective pixels that are correctly detected, i.e., the detection rate of ground-truth defect pixels, as shown in Equation (4):

(4) $${Recall}_{C_{n}}\%=\frac{TP}{TP+FN}\times 100\%$$

These metrics are computed independently for each defect category to provide a detailed assessment of segmentation performance across different defect types.

#### 4.2.2. Calculation of Weighted Performance Metrics for Defects in Images

Because multiple defect categories may coexist within a single lock surface and their spatial proportions vary across images, weighted evaluation metrics were adopted to reflect the defect distribution characteristics. Therefore, performance metrics were first computed by determining the weight proportion of each defect category within an image, as shown in Equation (5). The weighted performance metrics were then calculated according to these proportions, as defined in Equations (6)–(9). The weight and the weighted evaluation metrics were computed as:

(1) Weight calculation:

(5) $$W_{C_{n}}\%=\frac{\sum \text{label\_mask}(C_{n})}{\sum_{n=1}^{3}\text{label\_mask}(C)}\times 100\%$$

(2) Weighted mean IoU:

(6) $$\mathrm{Weighted}\;\mathrm{mIoU}\%={\sum_{n=1}^{N}W_{C_{n}}\times IoU}_{C_{n}}\times 100\%$$

(3) Weighted mean Dice:

(7) $$\mathrm{Weighted}\;\mathrm{mDice}\%={\sum_{n=1}^{N}W_{C_{n}}\times Dice}_{C_{n}}\times 100\%$$

(4) Weighted Precision:

(8) $$\mathrm{Weighted}\;Precision\%={\sum_{n=1}^{N}W_{C_{n}}\times Precision}_{C_{n}}\times 100\%$$

(5) Weighted Recall:

(9) $$\mathrm{Weighted}\;Recall\%=\sum_{n=1}^{N}W_{C_{n}}\times {Recall}_{C_{n}}\times 100\%$$

This weighting strategy ensured that the final evaluation metrics proportionally reflected the relative severity and spatial dominance of each defect type within an image, thereby providing a more representative assessment of segmentation performance under multi-defect conditions.

#### 4.2.3. Calculation of Weighted Performance Metrics Based on Boundary Evaluation

Because the segmentation results in this study were evaluated at the pixel level, slight spatial deviations along object boundaries, often caused by manual annotation uncertainty or minor prediction offsets, can disproportionately affect quantitative metrics. To address this issue, a boundary-based weighted evaluation strategy was adopted. This approach relaxes strict pixel-wise matching by incorporating a tolerance region around object boundaries, thereby reducing sensitivity to minor boundary misalignments.

**Figure 18:**
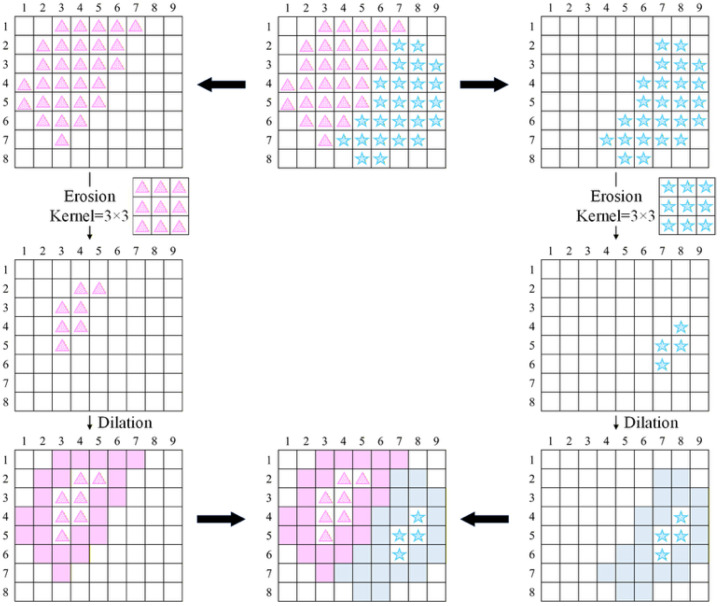
Illustration of boundary extraction and morphological operations for defining the ambiguous boundary region. Pink triangles and blue stars represent two adjacent regions or defect classes. The erosion operation (kernel = 3 × 3) shrinks each region, while the subsequent dilation expands it. Pink and blue shaded areas indicate the dilated regions of each class, and their overlapping region defines the ambiguous boundary. Arrows indicate the processing flow of morphological operations.

(1) Boundary extraction:

Let *M* denote a binary mask corresponding to a specific defect category. Boundary pixels are extracted using a morphological erosion operation:

*Erosion* = *erode*(*M*); *B* = *M* − *erode*(*M*) (10)

where *B* represents the boundary region obtained by subtracting the eroded mask from the original mask. As illustrated in Figure 18, erosion with a 3 × 3 structuring element first shrinks the defect region inward. The difference between the original mask and the eroded mask isolates the contour pixels, forming a one-pixel-thick boundary band. This process is applied to both the predicted mask and the ground-truth mask, enabling boundary-level comparison rather than strict region-level overlap.

(2) Determination of the tolerance radius τ:

To account for annotation and prediction uncertainties, a tolerance radius τ is defined relative to the image scale. First, the image diagonal length *D* is computed:

(11) $$D=\sqrt{H^{2}+W^{2}}$$

where *H* and *W* denote the image height and width, respectively. The tolerance radius is then calculated as:

(12) $$\tau =max\;[1,round\left(\alpha_{TR}\times D\right)]$$

where *α_TR_* is a predefined tolerance ratio, and the operator max (1,·) ensures that the tolerance radius is at least one pixel, preventing degenerate cases for small images. For an input image of size 256 × 256, *H* = 256 and *W* = 256, this results in $D=\sqrt{256^{2}+256^{2}}\;$≈ 362. The corresponding tolerance ratio $\alpha_{TR}$ can be determined for different values of τ: for *τ* = 2, $\alpha_{TR}\;$= 2/362 ≈ 0.0055; for *τ* = 3, $\alpha_{TR}\;$= 3/362 ≈ 0.0083; for *τ* = 4, $\alpha_{TR}\;$= 4/362 ≈ 0.0110. In this study, *τ* = 2 was selected based on the validation results, providing a suitable balance between tolerance coverage and boundary precision. This formulation scales the tolerance region proportionally to image size, ensuring consistent evaluation behavior across different resolutions.

(3) Construction of the boundary tolerance region:

After determining τ, morphological dilation is applied to the extracted boundary using an elliptical structuring element:

(13) kernel size=(2τ+1)×(2τ+1)

**Figure 19:**
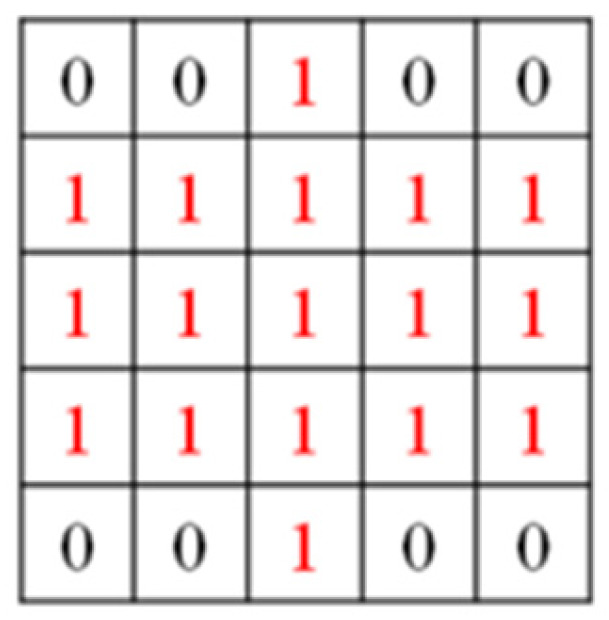
Elliptical kernel (5 × 5) for dilation. Red “1” indicates foreground (active elements in the structuring kernel), while black “0” denotes background.

As shown in Figure 19, when τ = 2, the resulting elliptical kernel size is 5 × 5. The use of an elliptical kernel ensures isotropic expansion of the boundary region, minimizing directional bias compared with rectangular kernels. This dilation produces a tolerance band surrounding the original contour. During evaluation, predicted boundary pixels falling within this tolerance band are considered acceptable matches to the ground-truth boundary. This boundary-relaxed evaluation strategy provides a more robust assessment for metallic lock defects, where edges are often reflective, irregular, and sensitive to small spatial deviations.

### 4.3. Optimal Parameter Configuration and Training Strategy

To ensure reliable model performance under limited data conditions, this study adopted a structured training and optimization strategy encompassing dataset design, augmentation, hyperparameter tuning, and resolution analysis. The dataset comprised 1200 images across three defect categories (scratch, white smoke, and bump damage) and was divided into 960 training, 120 validation, and 120 testing samples. All images were uniformly resized to 256 × 256 pixels to ensure architectural compatibility and consistent computational cost. Model selection and parameter optimization were conducted using the boundary-based F1-score, which emphasizes segmentation accuracy in defect boundary regions.

#### 4.3.1. Data Augmentation and Annotation Protocol

To address the limited dataset size and reduce the risk of overfitting, a comprehensive data augmentation strategy was applied during training. The augmentation pipeline included: (1) geometric transformations, including random rotation within ±15°, horizontal and vertical flipping, and scaling in the range of 0.9–1.1; (2) photometric variations, including brightness and contrast adjustments within ±20% to account for illumination variability; and (3) noise modeling, including Gaussian noise injection to emulate sensor noise and surface reflectance disturbances. These augmentations were applied online during training, effectively increasing the data diversity while preserving the structural characteristics of defects.

Given the inherent ambiguity of defect boundaries on reflective metallic surfaces, a rigorous annotation protocol was established. Three domain experts independently performed pixel-level annotations of defect regions. A consensus-based refinement process was then conducted to resolve discrepancies. To quantify annotation reliability, Cohen’s kappa coefficient was computed, yielding κ = 0.83, which indicates strong inter-annotator agreement. This ensures the robustness and consistency of the ground-truth labels used for model training and evaluation.

#### 4.3.2. Hyperparameter Optimization of U-Net-Based Models

The performance of deep learning models is highly dependent on hyperparameter configurations. In this study, key parameters, including training epochs, batch size, and learning rate, were systematically optimized using the boundary F1-score as the evaluation criterion. Because different network architectures exhibit distinct convergence behaviors and optimization dynamics, hyperparameter tuning was conducted independently for the baseline U-Net and two variants to ensure fair and architecture-specific optimization.

Hyperparameter optimization was conducted for U-Net, Res-U-Net, and U-Net++ to determine the optimal training configuration. The default settings were 200 epochs, a batch size of 2, and a learning rate of 0.0001. Sequential tuning experiments were performed by varying epochs, batch size, and learning rate, and model performance was compared using the boundary F1-score. For training duration, epoch values of 200, 400, and 600 were evaluated. All three models achieved superior segmentation performance at 400 epochs, which was therefore adopted as the final setting. Batch sizes of 1, 2, and 4 were tested under hardware constraints. The optimal batch size was 2 for U-Net, 4 for Res-U-Net, and 2 for U-Net++. Learning rates of 0.0005, 0.0001, and 0.00005 were initially assessed. Both U-Net and Res-U-Net achieved optimal performance at 0.0001. For U-Net++, performance differences between 0.0001 and 0.0005 were marginal; additional experiments with 0.001 and 0.005 were therefore conducted, revealing that 0.001 yielded the best segmentation performance.

The finalized hyperparameter configurations for each segmentation model are summarized in Table 1. The optimal configurations were determined independently for each architecture and subsequently adopted for all comparative experiments. Specifically, U-Net and Res-U-Net achieved optimal performance with 400 training epochs and a learning rate of 0.0001, with batch sizes of 2 and 4, respectively. U-Net++ also converged optimally at 400 epochs with a batch size of 2, but required a higher learning rate of 0.001. To ensure a controlled comparison and isolate the contribution of attention mechanisms, TransU-Net and FRCU-Net adopted the optimized hyperparameter configuration of the baseline U-Net.

#### 4.3.3. Effect of Input Resolution on Performance–Efficiency Trade-Off

To investigate the trade-off between segmentation accuracy and computational efficiency, experiments were conducted using five input resolutions ranging from 128 × 128 to 384 × 384 pixels. As shown in Table 2, increasing the input resolution improves the segmentation performance by preserving fine defect details, particularly for small and low-contrast defects. However, the performance gain becomes marginal beyond 256 × 256. Specifically, increasing the resolution from 256 × 256 to 384 × 384 yielded only a 0.48% improvement in F1-score while increasing the training time by approximately 61% and inference time by more than 50%. Conversely, reducing the resolution to 128 × 128 significantly degrades the performance due to the loss of critical defect features. These results indicate that excessively low resolution compromises detection capability, whereas excessively high resolution introduces unnecessary computational overhead with limited accuracy gains.

Therefore, 256 × 256 was selected as the optimal input resolution, providing a balanced trade-off between segmentation accuracy and computational efficiency. This resolution ensures sufficient preservation of defect characteristics while maintaining practical inference speed for industrial deployment.

### 4.4. Effectiveness and Efficiency Comparison of Metallic Lock Surface Defect Inspection

This study evaluated the segmentation performance and computational efficiency of five models: U-Net, Res-U-Net, U-Net++, TransU-Net, and FRCU-Net. Each model was trained with its optimal hyperparameter configuration, while TransU-Net and FRCU-Net adopted U-Net settings to isolate the effects of attention mechanisms. For practical reference, a human-inspection baseline was also evaluated using the same test dataset. Five experienced inspectors with domain expertise in metallic surface defect inspection manually annotated defect regions under consistent viewing conditions. All inspectors performed manual annotation on the same 2D digital images displayed on a monitor, rather than inspecting physical components directly. This setup ensured a fair and controlled comparison between human inspectors and the deep learning model, as both operate on identical visual inputs.

Segmentation performance was evaluated using pixel-wise F1-score, Precision, and Recall for each defect category. Given the pronounced class imbalance and the small, scattered nature of defect regions, conventional metrics such as Classification Rate (CR) are inappropriate because they are biased toward the majority class. Although Intersection over Union (IoU) is widely used in semantic segmentation, it may inadequately capture performance for defects with irregular boundaries and boundary sensitivity. Therefore, performance was assessed using Precision, Recall, and F1-score under a weighted boundary-based evaluation framework, which better reflects segmentation quality for small, irregular defects and reduces annotation uncertainty.

All experiments were conducted over five independent runs with different random seeds, and the results are summarized in Table 3. Among all models, TransU-Net achieved the best overall performance, with an F1-score of 85.15% ± 0.84%, outperforming U-Net (81.32%), U-Net++ (79.67%), FRCU-Net (81.04%), and human inspection (80.34%). It also maintained balanced Precision (83.94%) and Recall (86.41%), indicating superior capability in detecting defect boundaries and irregular structures. In contrast, Res-U-Net exhibited the lowest F1-score (76.25%), despite having the shortest training time and inference time.

All inference experiments were conducted with a batch size of 1 to reflect real-time deployment conditions. The reported inference time corresponds solely to the model forward pass and excludes preprocessing steps such as Canny edge detection, Hough transform, ROI masking, and image resizing. In terms of computational efficiency, U-Net provided a strong baseline with moderate training time (8210.52 s) and low inference latency (0.2183 s/image). Although TransU-Net introduced additional computational overhead due to Transformer-based self-attention, its inference time (0.3357 s/image) remained within a practical range while delivering the highest segmentation accuracy. FRCU-Net, despite achieving competitive precision, required the longest training time (10,362.76 s) and exhibited lower recall, indicating less balanced performance.

These results suggest that Transformer-based global attention is particularly effective for modeling geometric continuity and boundary characteristics, which dominate metallic lock defects. In contrast, frequency-based attention (FRCU-Net) emphasizes texture information and is less suited for boundary-dominant defect patterns. Therefore, the choice of attention mechanism should be guided by the structural characteristics of the target defects to achieve an optimal balance between accuracy and efficiency.

Table 4 presents the results of paired *t*-tests comparing the segmentation performance among different models and the human inspection baseline. The proposed TransU-Net model demonstrated statistically significant improvements over U-Net, FRCU-Net, and human inspection across all evaluation metrics, with *p*-values below 0.05, confirming the robustness of its performance gains. In particular, the improvement in F1-score between TransU-Net and U-Net (*p* = 0.002) indicates that the observed accuracy enhancement is not due to random variation. Similarly, TransU-Net significantly outperformed human inspection, reflecting both higher accuracy and greater consistency. In contrast, comparisons between U-Net, FRCU-Net, and human inspection showed limited statistical significance in most cases (*p* > 0.05), suggesting comparable performance among these approaches. In addition, at the 95% confidence level, the proposed TransU-Net improved the F1-score over the baseline U-Net by approximately 2.46–5.20%, indicating that the performance gain is both statistically significant and practically meaningful for industrial defect inspection. Overall, these results validate that the integration of Transformer-based attention leads to statistically significant and reliable improvements in defect segmentation performance.

The human inspectors achieved an F1-score of 80.34% ± 2.02%, with relatively higher variability compared to deep learning models, reflecting the subjective nature of manual inspection and the difficulty of delineating ambiguous defect boundaries on reflective surfaces. In comparison, TransU-Net demonstrated both higher accuracy and greater consistency. In terms of efficiency, manual inspection required approximately 5–10 s per sample, whereas model inference required 0.3357 s per image (excluding acquisition and preprocessing). These results indicate that the proposed system has strong potential to improve both inspection consistency and throughput in industrial applications.

### 4.5. Robustness and Component Contribution Analysis of the Proposed Method

To comprehensively evaluate the reliability and effectiveness of the proposed segmentation framework, supplementary analyses were conducted from two complementary perspectives: parameter sensitivity and component contribution. First, the impact of tolerance parameter settings on boundary-aware segmentation performance was examined to assess the stability of the evaluation framework under varying conditions. Second, an ablation study was performed to quantify the contribution of key system components. Together, these analyses provide a systematic assessment of the framework’s robustness, interpretability, and consistency in performance.

#### 4.5.1. Impact of Tolerance Numerical Differences on the Effectiveness of Image Segmentation

**Figure 20:**
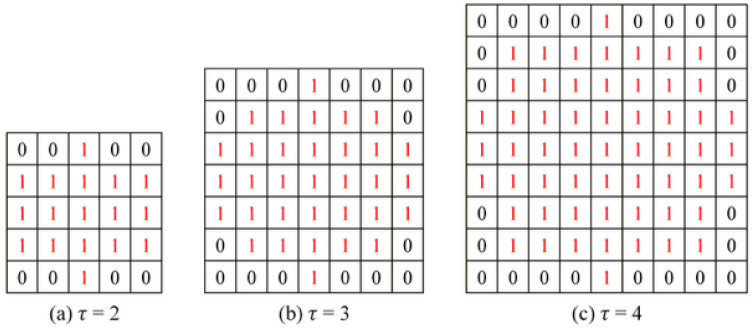
Dilation structuring elements for three tolerance values. Red “1” indicates foreground (active structuring element), while black “0” denotes background.

In the boundary-relaxed evaluation framework, the tolerance parameter τ controls the extent of the ambiguous boundary region and therefore directly influences the weighted performance metrics. Specifically, τ determines the size of the dilation structuring element according to Equation (13), thereby defining the spatial allowance for boundary deviations. To systematically analyze the sensitivity of segmentation performance to tolerance selection, three tolerance values (τ = 2, 3, 4) were evaluated. As shown in Figure 20, substituting different tolerance values into the kernel formulation produces the elliptical structuring elements with sizes 5 × 5, 7 × 7, and 9 × 9. The elliptical kernel ensures isotropic boundary expansion, preventing directional bias during dilation. Increasing τ enlarges the dilation radius and consequently broadens the ambiguous boundary band. If τ = 1, the resulting 3 × 3 cross-shaped kernel provides minimal expansion and limited tolerance for boundary shifts.

Table 5 presents the complete boundary extraction sequence under different tolerance values, including erosion, dilation, and ambiguous region construction. The eroded region represents high-confidence interior pixels with minimal annotation uncertainty. The dilated boundary expands outward from the contour. The ambiguous region is defined as the area between the eroded and dilated boundaries and is visualized in semi-transparent yellow. As τ increases, the ambiguous region expands substantially. While this reduces strict boundary mismatch penalties, excessive tolerance progressively deviates from the true annotated contour, potentially overestimating segmentation quality. From visual inspection, τ = 2 (5 × 5 kernel) produces an ambiguous band that closely follows the original boundary geometry without excessive expansion.

It should be noted that the tolerance parameter τ was determined exclusively using the validation set to avoid data leakage. The test set was strictly reserved for final performance evaluation, and no parameter tuning was performed on the test data. Table 6 reports the corresponding quantitative results under different tolerance settings. A clear trend was observed: increasing τ leads to higher F1-score and Recall values, and IoU decreases as the tolerance region expands excessively. This phenomenon occurs because larger tolerance regions incorporate more mismatched boundary pixels into the acceptable evaluation zone. As the ambiguous band widens, more prediction deviations are treated as correct, artificially inflating certain metrics, particularly Recall. Specifically, τ = 2 yielded the highest IoU (85.88%), indicating strong structural consistency with minimal over-relaxation; τ = 3 and τ = 4 increased the F1-score but reduced IoU, suggesting excessive boundary tolerance and potential metric inflation. Therefore, although larger tolerance values appear to improve certain performance indicators, they introduce evaluation bias by permitting greater boundary deviation.

An appropriate tolerance parameter should balance: compensation for annotation uncertainty on reflective metallic surfaces, preservation of structural fidelity to ground-truth boundaries, and prevention of artificial metric inflation. Based on both visual boundary conformity (Table 5) and quantitative stability (Table 6), τ = 2 provides the most reasonable trade-off. It maintained a geometrically consistent ambiguous region while achieving the highest IoU (85.88%), thereby ensuring robust yet conservative boundary-aware evaluation.

#### 4.5.2. Ablation Analysis of Component Contributions in the Proposed Inspection Framework

To systematically evaluate the contribution of individual components in the proposed inspection framework, an ablation study was conducted, as summarized in Table 7. The analysis investigated the incremental impact of three key elements: controlled image acquisition (ring illumination and rotational fixture), ROI masking, and the Transformer-based attention mechanism (TransU-Net).

Starting from the baseline U-Net model without enhancements, segmentation performance was limited by strong surface reflectivity and background interference. By introducing controlled image acquisition, the F1-score increased significantly, demonstrating that stabilized illumination and consistent viewing geometry effectively reduce reflectivity-induced noise and improve feature consistency. The addition of ROI masking further enhanced performance by eliminating irrelevant background regions and focusing the model on defect-prone areas. This led to noticeable improvements in both precision and recall, indicating more accurate and stable defect localization.

Finally, incorporating the Transformer-based attention mechanism (TransU-Net) yielded a substantial performance gain. This improvement was attributed to the model’s ability to capture long-range dependencies and global contextual information, which is particularly beneficial for detecting irregular defect structures and refining ambiguous boundaries on reflective surfaces. The proposed full framework improved the F1-score from 48.46% to 85.15%, corresponding to an absolute gain of 36.69%, clearly demonstrating the effectiveness of the integrated hardware and software design. Overall, the results demonstrate that each component contributes positively to segmentation performance, and their integration produces a synergistic effect, significantly improving both accuracy and robustness compared to the baseline model.

### 4.6. Discussion and Limitations

The experimental results demonstrate that integrating controlled image acquisition with a Transformer-enhanced segmentation model significantly improves the reliability of metallic lock surface defect inspection. The standardized rotational fixture and ring illumination reduce positional and reflective variability, providing stable and consistent inputs for learning-based segmentation. The TransU-Net architecture further enhances performance by capturing global contextual information and refining ambiguous defect boundaries, particularly under low-contrast and reflective conditions. In addition, the boundary-aware weighted evaluation scheme offers a more realistic assessment of segmentation performance by explicitly accounting for annotation uncertainty in boundary regions.

From an imaging perspective, alternative optical approaches such as polarization filtering and anti-reflective coatings are commonly used to suppress specular reflections in metallic surface inspection. Although polarization-based imaging can effectively reduce glare, prior studies have shown that its effectiveness degrades for objects with complex geometry and spatially varying surface properties, where no single polarization configuration can uniformly suppress reflections across all viewing angles [32,33]. Anti-reflective coatings (ARCs) are also widely applied to reduce surface reflections and enhance optical transmission; however, their performance is often influenced by durability, environmental conditions, and application constraints in industrial settings, particularly under high-throughput or contact-sensitive workflows [34,35,36]. In contrast, the proposed rotational acquisition strategy captures multi-view information, allowing defects obscured by specular reflection in one view to be revealed in another. This design improves robustness to reflectance variability without requiring surface modification or precise optical alignment. Although it introduces additional mechanical complexity, the approach provides a more generalizable and non-invasive solution for reflective metallic inspection under practical industrial conditions.

In addition to imaging system design and algorithmic improvements, surface manufacturing and post-processing techniques can also significantly influence inspection performance. For example, laser-based fabrication and finishing processes, such as laser powder bed fusion and laser polishing, can reduce surface roughness and improve surface uniformity, thereby reducing specular reflection and enhancing defect visibility. Liović et al. [37] demonstrated that laser-based processing can effectively control surface characteristics, leading to more homogeneous textures. Similarly, laser polishing has been shown to significantly improve surface smoothness and optical consistency [38,39], thereby benefiting vision-based inspection. These findings suggest that integrating manufacturing optimization with inspection systems can further enhance detection reliability. However, such approaches may introduce additional cost and process complexity. Therefore, the proposed framework focuses on improving robustness under existing industrial conditions without modifying the manufacturing pipeline, while recognizing that combining both strategies represents a promising direction for future work.

**Figure 21:**
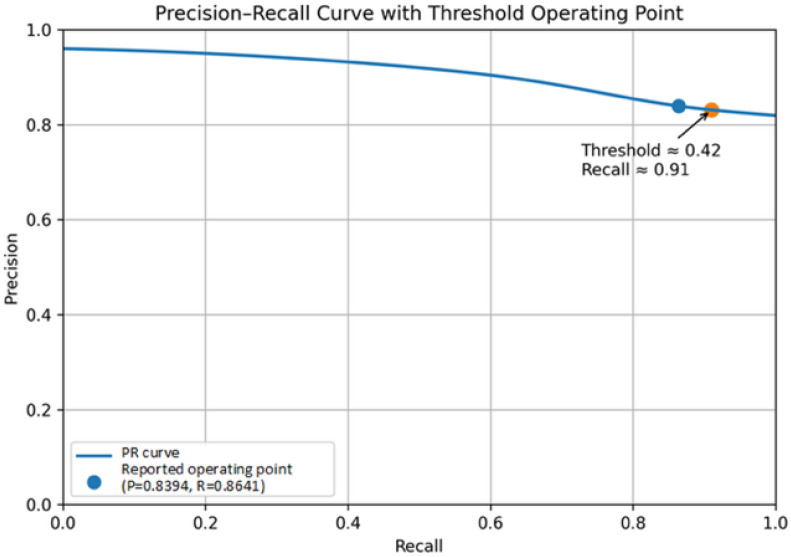
Precision–Recall curve of the proposed TransU-Net model. The marked point shows the reported operating point, while the annotated point (threshold ≈ 0.42) indicates a high-Recall setting (Recall ≈ 0.91), illustrating the adjustable Precision–Recall trade-off for industrial inspection.

To further evaluate the trade-off between detection sensitivity and precision, a Precision–Recall (PR) curve was generated by varying the decision threshold of the TransU-Net model. As shown in Figure 21, the model maintained relatively stable Precision across a broad range of Recall values, demonstrating robust discriminative capability under different operating conditions. Importantly, higher Recall can be achieved with only a moderate reduction in Precision, which is particularly desirable in industrial inspection scenarios where minimizing false negatives (i.e., missed defects) is critical. This analysis complements the F1-score by providing a more comprehensive characterization of model performance across different decision criteria.

The decision threshold, applied to the pixel-wise probability map after the sigmoid activation of the TransU-Net model, enables flexible control of the Precision–Recall trade-off without retraining the model. As illustrated in Figure 21, setting the threshold to 0.42 enabled the model to achieve a high Recall level (≈91%), thereby reducing the likelihood of undetected defects. Although this may increase false positives, such a trade-off is generally acceptable in industrial quality control, where the cost of missed defects outweighs over-rejection. This thresholding flexibility allows the proposed system to be readily adapted to different industrial risk preferences and deployment requirements.

To clarify the distinction between algorithmic inference speed and practical deployment efficiency, Table 8 summarizes the estimated system-level takt-time of the proposed inspection framework. The previously reported value of 0.3357 s corresponds only to model inference for a single image and does not represent the full inspection cycle. In the complete workflow, additional time is required for part positioning, multi-view image acquisition, stepper-motor rotation, ROI preprocessing, and decision output. Under a purely sequential workflow, the estimated end-to-end inspection cycle is approximately 2.44 s per lock. Although these values remain higher than the model-only inference time, they demonstrate that system-level optimization can substantially improve throughput while preserving the accuracy benefits of the proposed TransU-Net-based inspection framework.

To provide a more realistic projection of deployment performance, it is important to note that the reported inference time in Table 8 is based on the sequential processing of individual views. In practical implementation, the four cropped images acquired from different viewing angles can be processed simultaneously using GPU batch inference (e.g., batch size = 4). Given the parallel processing capability of the NVIDIA RTX 3060, this approach can reduce the effective model inference time from approximately 1.34 s to around 0.35 s per component. This optimization requires no modification to the trained model and can be directly integrated into the deployment pipeline. However, the overall system cycle time remains constrained by image acquisition and mechanical actuation, indicating that further efficiency improvements should focus on hardware-level optimization.

Despite these advantages, several limitations should be noted. First, the dataset size was relatively limited and focused on a specific lock type, which may restrict generalization to other metallic components with different geometries or surface finishes. Second, the imaging setup was evaluated under controlled conditions, and its performance under more variable industrial environments, such as changes in ambient lighting or surface contamination, requires further investigation. Third, although the TransU-Net model improves segmentation accuracy, it introduces additional computational complexity compared with conventional CNN-based architectures, which may require optimization for deployment on resource-constrained edge devices. Fourth, while the proposed system demonstrated improved inspection consistency, the complete system-level cycle time includes image acquisition, mechanical rotation, and preprocessing, which are not reflected in the model inference time alone and should be considered in practical deployment.

A limitation of the proposed framework lies in the reliance on classical computer vision techniques, specifically Canny edge detection and Hough transform, for ROI extraction before model inference. While effective under controlled laboratory conditions, these deterministic methods may be sensitive to illumination variations, such as gradual degradation of LED ring-light intensity or changes in environmental lighting in industrial settings. As a result, the system may fail at the ROI extraction stage before the TransU-Net model is engaged, potentially affecting overall inspection reliability. To improve robustness, future work will investigate more reliable strategies, including adaptive thresholding, periodic calibration of the imaging system, and learning-based ROI detection approaches. These improvements aim to enhance system stability and ensure reliable operation under long-term industrial deployment conditions.

Finally, although the comparative analysis in this study was primarily conducted within the U-Net family to ensure a controlled evaluation of architectural modifications under limited-data conditions, comparisons with non-U-Net state-of-the-art architectures could provide additional insights. Such models typically require larger datasets and different optimization strategies, and are therefore considered beyond the scope of this study. Future work will focus on expanding dataset diversity, evaluating cross-domain generalization across different factories and surface finishes, incorporating additional segmentation architectures, and optimizing system integration for real-time industrial applications.

## 5. Conclusions

This study presented an integrated automated visual inspection framework for metallic lock surface defects, addressing challenges associated with strong reflectivity, low contrast, and structural variability. A standardized rotational fixture and a controlled illumination system were developed to stabilize imaging geometry and ensure consistent image acquisition. In addition, a customized ROI masking strategy effectively suppressed background interference, enabling more focused and reliable feature learning. At the algorithmic level, a Transformer-enhanced U-Net architecture integrates global contextual modeling with convolutional feature extraction, improving defect boundary delineation and detection of irregular surface anomalies. Furthermore, a boundary-aware weighted evaluation scheme was introduced to provide a more realistic assessment of segmentation performance under low-contrast and ambiguous boundary conditions.

Experimental results on production-line samples demonstrate that the proposed TransU-Net model achieved an F1-score of 85.15%, with an average inference time of 0.3357 s per image. These results indicate improved inspection accuracy and consistency compared with manual visual inspection. The findings confirm that the integration of controlled image acquisition and advanced segmentation architectures provides an effective and scalable solution for automated inspection of reflective metallic surfaces. Future work will focus on expanding dataset diversity across different metallic components and surface conditions, improving robustness under varying industrial environments, and optimizing computational efficiency for real-time deployment on embedded and edge computing platforms.

## Figures and Tables

**Table 1:** Summary of the optimal parameter settings for each model used in this study.

Model	Training Epoch	Batch Size	Learning Rate
U-Net	400	2	0.0001
Res-U-Net	400	4	0.0001
U-Net++	400	2	0.001
TransU-Net	U-Net configuration adopted		
FRCU-Net			

**Table 2:** Effect of input resolution on segmentation performance and computational efficiency using the TransU-Net method.

Input Image	F1-Score (%)	Precision (%)	Recall (%)	Total Training Time (s)	Inference Time (s/Image)
128 × 128	80.21 ± 1.32	79.12 ± 1.45	81.36 ± 1.62	4758	0.2145
192 × 192	82.22 ± 1.05	81.05 ± 1.18	83.47 ± 1.26	6123	0.2763
256 × 256	85.15 ± 0.84	83.94 ± 0.87	86.41 ± 0.95	7780	0.3357
320 × 320	85.48 ± 0.79	84.26 ± 0.82	86.73 ± 0.90	9874	0.4216
384 × 384	85.63 ± 0.76	84.41 ± 0.79	86.85 ± 0.88	12,552	0.5128

**Table 3:** Comparison of segmentation effectiveness and computational efficiency of U-Net and its variants under the weighted boundary-based evaluation framework, including a human inspection baseline.

Method	F1-Score (%)	Precision (%)	Recall (%)	Total Training Time (s)	Inference Time (s/Image)
U-Net	81.32 ± 1.08	79.90 ± 1.12	82.64 ± 1.35	8210.52 ± 61.57	0.2183 ± 0.0001
Res-U-Net	76.25 ± 0.96	79.03 ± 1.05	78.47 ± 1.18	2112.14 ± 25.26	0.2070 ± 0.0001
U-Net++	79.67 ± 0.92	80.92 ± 0.98	82.21 ± 1.10	4742.07 ± 34.76	0.2213 ± 0.0001
TransU-Net	85.15 ± 0.84	83.94 ± 0.87	86.41 ± 0.95	7780.17 ± 55.13	0.3357 ± 0.0003
FRCU-Net	81.04 ± 0.95	87.74 ± 0.79	76.02 ± 1.10	10,362.76 ± 67.73	0.2740 ± 0.0002
Human inspection	80.34 ± 2.02	82.15 ± 2.10	78.62 ± 2.45	---	5~10 per sample

**Table 4:** Paired *t*-test results for model comparisons (*p*-values).

Model Comparison	F1-Score (*p*-Value)	Precision (*p*-Value)	Recall (*p*-Value)
U-Net vs. TransU-Net	0.002	0.002	0.004
U-Net vs. FRCU-Net	0.413	0.001	0.003
U-Net vs. human inspection	0.356	0.129	0.094
TransU-Net vs. FRCU-Net	0.001	0.001	0.001
TransU-Net vs. human inspection	0.002	0.002	0.004
FRCU-Net vs. human inspection	0.216	0.021	0.068

**Table 5:** Effect of using various tolerance values on the identification of ambiguous regions in images. The color encoding of defect categories is consistent with the RGB definitions provided in Figure 8.

	*τ* = 2, Kernel 5 × 5	*τ* = 3, Kernel 7 × 7	*τ* = 4, Kernel 9 × 9
Erosion process	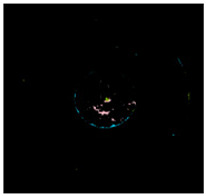	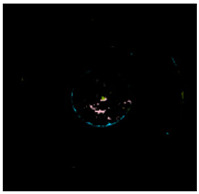	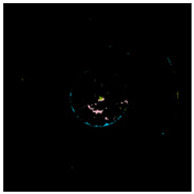
Dilation process	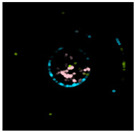	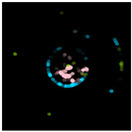	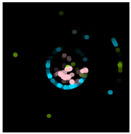
Ambiguous regions	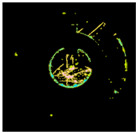	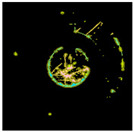	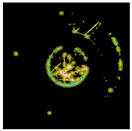

**Table 6:** Comparisons of different tolerance values on image segmentation effectiveness.

Tolerance Value	IoU	F1-Score	Precision	Recall
*τ* = 2, 5 × 5	85.88%	81.32%	79.90%	87.41%
*τ* = 3, 7 × 7	69.97%	85.12%	84.33%	89.64%
*τ* = 4, 9 × 9	56.88%	87.56%	87.10%	91.22%

**Table 7:** Ablation study on the contribution of key components in the proposed inspection framework.

Configuration	Controlled Acquisition (Lighting + Rotation)	ROI Masking	Transformer (TransU-Net)	F1-Score (%)	Precision (%)	Recall (%)
(A) Baseline U-Net	✗	✗	✗	48.46 ± 1.21	46.85 ± 1.34	50.21 ± 1.48
(B) + Controlled Acquisition	✓	✗	✗	70.12 ± 1.05	68.94 ± 1.12	71.45 ± 1.29
(C) + ROI Masking	✓	✓	✗	81.32 ± 1.08	79.90 ± 1.12	82.64 ± 1.35
(D) + Transformer (Full Model)	✓	✓	✓	85.15 ± 0.84	83.94 ± 0.87	86.41 ± 0.95

Note: “✓” indicates that the corresponding component is included in the configuration, “✗” indicates that it is not included, and “+” denotes the incremental addition of a component relative to the previous configuration.

**Table 8:** Estimated system-level takt-time breakdown for the proposed automated lock surface inspection system.

Process Step	Description	Time per Image/View (s)	Views per Lock	Estimated Time (Sequential Inference) per Lock (s)	Estimated Time (Parallel Inference) per Lock (s)
Part positioning	Placement and stabilization of the lock sample in the fixture	—	—	0.20	0.20
Image acquisition	Camera exposure and image transfer	0.08	4	0.32	0.32
Mechanical rotation	Stepper-motor rotation to the next 90° position and settling time	0.10	3	0.30	0.30
ROI preprocessing	Cropping, masking, edge detection, and circle localization	0.05	4	0.20	0.20
Model inference	TransU-Net forward prediction	0.3357	4	1.34	0.35
Post-processing/decision	Aggregation of results and pass/fail decision	—	—	0.08	0.08
Total estimated cycle time	End-to-end inspection for one lock			2.44	1.45

## Data Availability

The data will be made available on request.
